# Interactions Between Immunosuppressive Regimens and Cytomegalovirus Infection After Solid-Organ Transplantation

**DOI:** 10.3389/ti.2026.15987

**Published:** 2026-04-09

**Authors:** Lucas Milo Bellier, Hannah Kaminski, Pierre Merville, Lionel Couzi

**Affiliations:** 1 Centre Hospitalier Universitaire de Bordeaux, Bordeaux, France; 2 UFR des Sciences Médicales, Universite de Bordeaux, Talence, France; 3 Immuno ConcEpT, CNRS-UMR 5164, Université de Bordeaux, Talence, France

**Keywords:** cytomegalovirus (CMV), immunosuppressant, infection, kidney transplant, review of literature

## Abstract

Cytomegalovirus (CMV) remains a major infectious complication after solid-organ transplantation, driven by immunosuppressive therapies that alter CMV-specific cell-mediated immunity. Antithymocyte globulin induces profound and prolonged T-cell depletion, transiently impairing CMV-specific cell-mediated immunity and increasing CMV risk in seropositive recipients. Calcineurin inhibitors suppress cytokine production, notably IL-2 and IFN-γ, without significantly impairing cytotoxic function, while mycophenolate mofetil limits lymphocyte proliferation but preserves effector capacity. In contrast, mTOR inhibitors exert dual antiviral and immunomodulatory effects by directly inhibiting CMV replication and enhancing CMV-specific T-cell memory formation. Belatacept, through CD28–CD80/CD86 blockade, may predispose to late, severe, or relapsing CMV disease, particularly in elderly or D^+^/R^−^ recipients. Corticosteroids broadly inhibit NK cell cytotoxicity and CMV-specific T-cell responses, but clinical data on steroid withdrawal remain inconsistent. Overall, CMV risk is determined less by a single drug than by the cumulative depth of immunosuppression. Integrating immune monitoring tools, such as CMV-specific T-cell assays, could enable tailored immunosuppressive regimens balancing antiviral protection with graft survival.

## Introduction

Cytomegalovirus (CMV) is a major opportunistic pathogen in solid-organ transplantation (SOT), where immunosuppressive therapy promotes viral reactivation and disease.

In immunocompetent hosts, early recognition of CMV by dendritic cells and macrophages through Toll-like receptors triggers type I interferon and cytokine production, activating natural killer (NK) cells and limiting initial viral replication.

Long-term viral control, however, relies on adaptive immunity, particularly CMV-specific CD8^+^ T cells. Upon antigen presentation, these cells expand and differentiate into effector and memory subsets that clear infected cells via cytotoxic mechanisms (perforin, granzymes) and cytokine release (IFN-γ, TNF-α). Persistent infection drives “memory inflation,” characterized by the progressive accumulation of highly differentiated, functional CD8^+^ T cells targeting dominant antigens (IE1, pp65) [1]. These cells are crucial for maintaining viral latency, and their deficiency correlates with uncontrolled replication in immunocompromised patients. Functional monitoring of CMV-specific T-cell responses can be achieved using intracellular cytokine staining (ICS), CMV-ELISPOT, or interferon-gamma release assays (e.g., QuantiFERON®-CMV), which differ in sensitivity and clinical applicability [2].

Although αβ CD8^+^ and CD4^+^ T cells dominate adaptive control, γδ T cells—especially nonVδ2Vg9 subsets—also expand during infection and exhibit cytotoxic, IFN-γ–producing effector functions, compensating when conventional T-cell immunity is impaired [3], together with NK CD57^+^ NKG2C (high) NK cells [4].

Understanding immune dynamics is critical to assess how immunosuppressive therapies influence CMV susceptibility and clinical outcomes. This review examines the impact of each immunosuppressant class on CMV risk, adopting a structured approach according to risk level.

## High-Risk Agents for CMV Infection

This section identifies immunosuppressive agents that substantially increase CMV infection risk through profound immunosuppression, requiring heightened vigilance and tailored management strategies.

### Antithymocyte Globulin: The Most Depleting Agent

#### Mechanisms of Action

Antithymocyte globulins (ATG) cause profound T-cell depletion by targeting multiple surface antigens. Their main mechanisms include complement-dependent lysis, apoptosis, and opsonization with phagocytosis. Beyond depletion, ATG modulate immune responses by downregulating adhesion molecules and chemokine receptors, disrupting leukocyte trafficking [5]. ATG induces rapid and profound depletion of naive CD4^+^ T cells, more pronounced than that observed for CD8^+^ T cells. Notably, CD4^+^ lymphopenia often persists at 12 months post-transplantation [6].

ATG has been widely associated with profound immunosuppression and an increased incidence of cytomegalovirus (CMV) infection [7–11]. Since its initial use, it has been recognized that ATG may promote CMV infection by depleting CMV-specific cell-mediated immunity (CMI) [12].

#### Impact According to Serological Status

According to the prospective multicenter observational study by Kumar et al., very few D+R- SOTRs exhibit detectable CMV-specific CMI, as measured by ELISPOT assay, at the time of transplantation [13]. However, this finding has been challenged by a single-center Spanish cohort study, which reported that approximately 25% of D^+^/R^−^ patients had detectable CMV-specific CMI at transplantation [14]. These differences may, at least in part, be explained by differences in CMV prevalence worldwide. Furthermore, most CMV-seronegative recipients do not develop a detectable T-cell response during the prophylactic period [12]. Consequently, at the end of prophylaxis, only a small proportion of D^+^/R^−^ SOTRs exhibit CMV-specific T-cell CMI [13]. Specifically, only 12.1% of D+R− patients have a positive QuantiFERON-CMV assay at the end of prophylaxis [15], a number that can rise to 20.9% when measured by ELISPOT assay [16]. Following these immunological observations, it has been demonstrated that ATG induction therapy, compared to anti-IL-2 receptor antagonists (IL-2RA), did not confer an additional risk of CMV DNAemia or disease in D^+^/R^−^ kidney transplant recipients [17]. As the increased risk of CMV associated with ATG is probably related to its depleting effect on CMV-specific T lymphocytes, this risk does not increase in patients who mostly lack CMV-specific CMI. Nevertheless, this treatment may exacerbate infection severity or recurrence rate, a possibility that requires further investigation.

**FIGURE 1:**
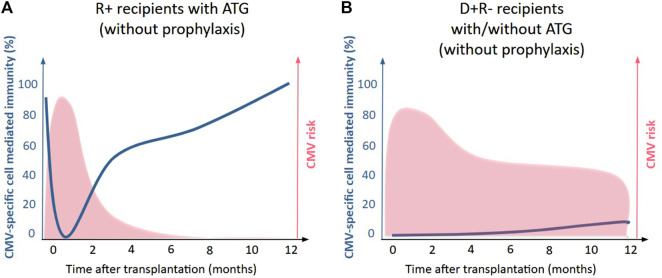
Effect of rabbit anti-thymocyte globulin (rATG) on CMV-specific immunity according to recipient serostatus. This figure is a schematic representation of the restoration of CMV- specific immunity. **(A)** In CMV-seropositive (R^+^) recipients, rATG transiently depletes CMV-specific T cells, increasing CMV disease risk until immune reconstitution occurs. **(B)**. In CMV-seronegative (D^+^/R^−^) recipients, CMV risk is high with or without rATG, as CMV-specific cell mediated immunity is largely absent at baseline.

In CMV-seropositive recipients (R+), most of the SOTRs have a CMV-specific CMI both at the time of transplantation and at the end of prophylaxis but this response is highly heterogeneous between the patients [13–18]. Consistent with these findings, it has been demonstrated that ATG induction therapy, compared with IL-2RA, confers an additional risk of CMV DNAemia and disease exclusively in R+ kidney transplant recipients [17]. Specifically, ATG increased the risk of CMV DNAemia in R+ recipients with detectable pretransplant CMI while no difference was observed among R+ recipients lacking detectable CMI, highlighting the pivotal role of CMV-CMI in the prevention of CMV infection (Figure 1). Furthermore, longitudinal analysis of CMV-specific CMI kinetics revealed that R+CMI+ recipients receiving rATG experienced a more pronounced suppression of their pre-existing CMV-specific immunity during the first two months post-transplantation compared to those treated with anti–IL-2RA, whereas no significant impact was observed in R+ CMI- recipients.

#### Recovery of CMV-Specific Immunity

The recovery of CMV-specific CMI after ATG has been studied in few studies. At one-month post-transplantation, approximately 50% of R+ SOTRs receiving ATG recover a detectable CMV-specific CMI, as measured by the QuantiFERON-CMV assay [19]. By 3 months post-transplant using ELISPOT, CMV-specific immune recovery is comparable between ATG-treated and non–ATG-treated patients [17], and among patients with detectable pretransplant CMV-specific CMI, immune responses recovered by 6 months post-transplant to levels similar to those observed prior to transplantation [18].

Taken together, these findings suggest that ATG transiently impairs CMV-specific T-cell responses in R+ SOTRs, while allowing for subsequent immune reconstitution over time [12]. The transient loss of CMV-specific CMI in these patients is highly correlated with the risk of CMV disease. Few data are available about ATG effect on γδ T cells and NK cells.

### Belatacept: The Special Case of Late and Atypical Infections

#### Clinical Evidence

Belatacept, a costimulation blocker targeting the CD28–CD80/CD86 pathway, has demonstrated efficacy in preventing alloimmune responses while preserving renal function. Clinical trials showed that belatacept used from transplantation did not increase CMV risk compared to cyclosporine. In the BENEFIT and BENEFIT-EXT trials, CMV infection rates were similar (6%–9% and 11%–14%, respectively) between belatacept and cyclosporine groups [20, 21]. In a smaller trial, Mannon et al. [22] observed more CMV viremia in the belatacept arm (20.7%), but differences were not statistically significant. Similarly, Woodle et al. [23] found comparable CMV rates (∼11%) across tacrolimus and two belatacept regimens. Switching from Calcineurin inhibitors (CNIs) to belatacept did not also appear to increase CMV disease incidence. Budde et al. [24] and Divard et al. [25] reported no rise in CMV complications after conversion, even in high-risk patients.

On the opposite, several retrospective studies have highlighted an increased risk of CMV infection in patients receiving belatacept. Bertrand et al. reported a higher incidence of late-onset CMV disease, following conversion to belatacept [26]. A subsequent study by the same group confirmed that both CMV-seropositive (R^+^) and high-risk CMV D^+^/R^−^ recipients were affected [27]. Similarly, Chavarot et al. described an increased incidence of atypical, often severe CMV disease—sometimes with intestinal or ocular involvement—occurring several years after transplantation (median 43.6 months post-conversion) [28]. These infections were particularly common among elderly recipients (>70 years), patients with impaired graft function, or those who underwent late conversion to belatacept. Moreover, CMV infections in belatacept-treated recipients frequently relapse despite initial antiviral therapy, occasionally necessitating discontinuation of belatacept [29]. A U.S. cohort by Karadkhele and colleagues similarly found higher CMV disease rates in CMV high-risk patients on belatacept, reinforcing that this association is not restricted to specific geographic regions or clinical settings [30].

#### Pathophysiological Mechanisms

**FIGURE 2:**
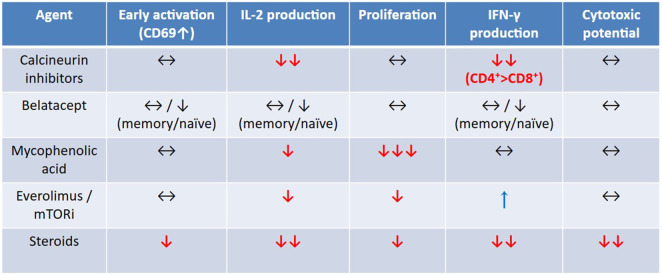
Simplified *in-vitro* effects of major immunosuppressive agents on CMV-specific T-cell functions. Assessed functions include early activation (CD69 upregulation), IL-2 production, proliferation, IFN-γ secretion, and cytotoxic potential (perforin/granulysin expression and target-cell killing). Data are derived from comparative *in vitro* studies using clinically relevant drug concentrations [31, 33, 51].

Xu et al. demonstrated that belatacept’s immunosuppressive effects are more pronounced in naïve T cells but attenuate as T cells mature [31]. Belatacept trough levels vary widely among kidney transplant recipients, ranging from 1.4 to 24.8 μg/L, with a mean of 8.4 ± 3.9 μg/L [32]. *In vitro*, belatacept demonstrates strong immunosuppressive effects on alloreactive T cells, with an IC50 of 4 ng/mL for IL-2 production [33]. However, CMV-specific T cells are less susceptible to belatacept’s antiproliferative effect compared to alloreactive T cells [31]. *In vivo*, CMV-specific CD4^+^ and CD8^+^ T-cell responses remain stable during the first year after switching to belatacept, with no significant decline in IFN-γ or TNF-α secretion [34] (Figure 2).

The immunopathogenesis of CMV reactivation under belatacept likely stems from its targeted blockade of the CD28^−^CD80/CD86 costimulatory pathway, which is essential for the activation of naïve T cells. Regarding antiviral function, belatacept reduces IFN-gamma production by 50% in peripheral blood mononuclear cells (PBMCs) stimulated with a CMV peptide pool [33]. This inhibition dampens the primary CMV-specific T cell response, a particularly critical defect in seronegative recipients of CMV-positive organs (D^+^/R^−^). Consistent with this, Kleiboeker et al. reported a case of failed CMV-specific cellular immunity development in a high-risk D^+^/R^−^ patient treated with belatacept in combination with low-dose tacrolimus, MPA, and steroids [35].

**FIGURE 3:**
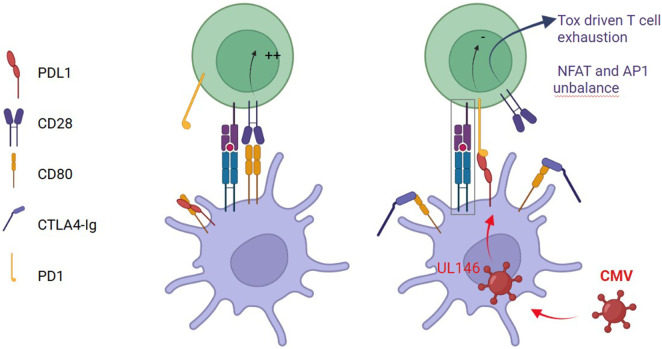
Proposed mechanisms by which belatacept promotes CMV-specific T-cell exhaustion. Belatacept disrupts CD80–PD-L1 cis interactions on antigen-presenting cells, increasing free PD-L1 and enhancing PD-1/PD-L1 inhibitory signaling. CMV amplifies this pathway via UL146-mediated PD-L1 upregulation. Downstream, NFAT/AP-1 unbalance promotes TOX-driven exhaustion of CMV-specific CD8^+^ T cells (adapted from [37]).

Belatacept does not impair the frequency of IL-2/TNF-α/IFN-γ triple-positive CD28^−^ memory CMV-specific T cells, indicating that cytokine production by these memory cells remains largely preserved [31]. However, beyond its effects on naïve T cells, belatacept may indirectly impair CMV-specific memory T cells that are CD28-independent. Under physiological conditions, CD80 interacts in cis with PD-L1 on antigen-presenting cells, thereby limiting PD-1/PD-L1 engagement. Because belatacept has a high binding affinity for CD80, it can disrupt CD80/PD-L1 heterodimers [36]. This displacement could release free PD-L1, enhancing trans interactions between PD-1 and PD-L1, and thereby promoting functional exhaustion of PD1-expressing CMV-specific CD8^+^ T cells [37]. CMV infection itself may further amplify this inhibitory axis. The viral UL146 gene product (vCXCL1) has been shown to upregulate PD-L1 expression on infected hepatic cells, thereby strengthening PD-1/PD-L1–mediated suppression [38]. Moreover, CD28 blockade alters downstream signaling balance between NFAT and AP-1, fostering TOX-driven transcriptional programs associated with T-cell exhaustion. Consistent with this mechanism, Long et al. demonstrated that abatacept—another CD28 pathway inhibitor—induced T-cell exhaustion, supporting a class effect of CD28 blockade [39]. Finally, rare human cases of inherited CD28 deficiency are characterized by susceptibility to chronic viral infections, providing genetic evidence of the central role of this pathway in antiviral immunity [40] (Figure 3).

#### Management Strategies

Given the increased CMV risk observed under belatacept, several strategies have been proposed to optimize its use while minimizing infection. In a recent study by Del Bello et al., conversion from MPA to an mTOR inhibitor in belatacept-treated kidney transplant recipients during or after a first CMV episode, led to a marked reduction in CMV replication or recurrence rates. In this study, the incidence of CMV DNAemia was 0.072 per month of exposure in patients treated with belatacept–MPA, compared with 0.035 in patients treated with belatacept–mTOR inhibitor (p = 0.0002) [41]. High-risk populations—particularly D^+^/R^−^ recipients, elderly patients—could also benefit early initiation of antiviral prophylaxis or pre-emptive therapy for at least 6 months following the initiation of belatacept therapy [26]. In the future, combining belatacept with agents that preserve CMV-specific immunity may help maintain the delicate balance between graft protection and antiviral defense [42].

## Neutral or Moderate Risk Agents

This section examines immunosuppressive agents that do not independently increase CMV risk when used as monotherapy, but may contribute to CMV susceptibility through cumulative immunosuppression when combined with other agents.

### Calcineurin Inhibitors (CNI): Tacrolimus and Cyclosporine

#### Mechanisms and Clinical Data

CNIs target specific cytoplasmic proteins—cyclophilins for cyclosporine and FK-binding proteins for tacrolimus—thereby inhibiting calcineurin, a key phosphatase involved in the activation of lymphocytes. This inhibition prevents the transcription of cytokines such as IL-2, TNF-α, and IFN-γ, which are critical for the immune response following alloantigen recognition.

CNIs remain the cornerstone of immunosuppressive therapy, and most CNI-sparing strategies have been compared to regimens based on mTOR inhibitors or belatacept. This makes it particularly challenging to isolate the specific effect of CNIs on the risk of CMV infection after transplantation. However, a few studies have attempted to assess the risk of CMV viremia and/or infection in KTRs undergoing CNI withdrawal compared to those maintained on CNI therapy. In the Cochrane meta-analysis by Karpe et al. [43], which reviewed 83 studies—including 30 that specifically reported on CMV-related disease incidence—a trend toward a protective effect of CNI withdrawal was observed. However, this trend did not reach statistical significance. Therefore, the clinical impact of CNI on CMV infection has not been definitively documented in clinical trials. Another important question is whether tacrolimus and cyclosporine have a similar impact on CMV infection. In the pivotal trials comparing the two CNIs, the incidence of CMV infection was found to be similar [44, 45]. In the SYMPHONY study, Ekberg et al. [46] reported CMV infection rates of 14.6% in the standard-dose cyclosporine group, 11% in the low-dose cyclosporine group, and 9.7% in the low-dose tacrolimus group. More recently, a retrospective comparison between the EVERCMV study and a *post hoc* analysis by Tedesco-Silva and colleagues showed comparable CMV DNAemia rates between patients treated with cyclosporine and those treated with tacrolimus, whether combined with everolimus (EVR) (39.2% vs. 43.3%) or with mycophenolic acid (MPA) (77.8% vs. 77.9%) [47, 48]. Additionally, the type of CNI (tacrolimus versus cyclosporine) was not associated with viral eradication in the VICTOR study [49]. These findings support the hypothesis that both the incidence and severity of CMV infection are largely independent of the type of CNI used.

#### Impact on CMV-Specific Immunity

CNIs have been shown to exhibit direct antiviral properties, including the ability to impair viral replication. A recent study demonstrated that CNIs exert antiproliferative effects on CMV *in vitro*, with a sixfold increase in anti-CMV activity when combined with MPA. However, these effects were observed at drug concentrations exceeding those typically achieved *in vivo* based on pharmacokinetic data [50].

Additionally, several studies have investigated the impact of CNI on CMV-specific lymphocyte responses. In a recent study by Krueger et al., low-dose tacrolimus (5 ng/mL) did not significantly reduce early activation of CD4^+^ and CD8^+^ T cells upon stimulation with CMV pp65, as measured by CD69 expression [51]. However, tacrolimus alone significantly suppressed IL-2 production by both CMV-specific CD4^+^ and CD8^+^ T cells. Tacrolimus showed high potency in suppressing IL-2, with an IC_50_ of approximately 1 ng/mL [33]. However, this IL-2 inhibition had little impact on the proliferative capacity of CMV-specific T cells, suggesting that another cytokine such as IL-15 could act as a substitute [51]. Cyclosporin has been tested on the proliferation capacity of both conventional CMV-specific and γδ T cells. A decreased proliferation capacity was observed at doses as low as 25 ng/mL [52].

Tacrolimus also reduces the production of IFN-γ, a key antiviral cytokine that limits CMV replication and dissemination. This effect is particularly pronounced in the CD4^+^ CMV-specific T cell subset [51]. Additional studies have confirmed that CNIs also suppress IFN-γ production in virus-specific memory CD8^+^ T cells [53]. Even at very low concentrations (1.5 ng/mL), tacrolimus was shown to reduce IFN-γ production by 80%–90% in CMV-specific T cells stimulated with CMV peptide pools for 72 h [33]. Furthermore, tacrolimus inhibits T cell responses in both CMV-naive and CMV-immune individuals [31]. However, tacrolimus does not appear to significantly impair cytolytic function, as it does not reduce the expression of perforin or granulysin in CMV pp65-stimulated PBMC [51].

Collectively, these findings highlight the nuanced impact of tacrolimus on CMV-specific T cell responses—sparing early activation and cytolytic potential, while strongly suppressing key cytokines like IFN-γ, which is essential for antiviral control (Figure 2).

### Mycophenolate Mofetil (MPA): Risk Related to Cumulative Effects

#### Mechanisms and Clinical Data

MPA acts as a noncompetitive, selective, and reversible inhibitor of inosine monophosphate dehydrogenase, the rate-limiting enzyme in *de novo* guanosine nucleotide synthesis [54]. Because lymphocytes depend exclusively on this pathway for purine synthesis, MPA profoundly inhibits T- and B-cell proliferation while sparing other cell types that can utilize salvage pathways.

In patients with lupus nephritis, MPA therapy showed no higher incidence of CMV infection compared to cyclophosphamide or azathioprine, both during induction and maintenance phases [55, 56]. The absence of CMV disease in these patients likely reflects the absence of allograft-derived viral reintroduction or post-transplant–specific “hits,” such as ischemia–reperfusion injury, which are known triggers of CMV infection [57]. In renal transplantation, early landmark trials found no significant increase in CMV infection with MPA/cyclosporine regimens compared with azathioprine or placebo [58–60]. Later, Miller et al. reported that MPA (1–2 g/day) in combination with tacrolimus did not result in higher CMV infection rates than azathioprine-based therapy [61]. In the VICTOR trial, the use of MPA had no significant influence on viral eradication [49]. More recent retrospective analyses confirm that MPA was not an independent risk factor for CMV recurrence or late CMV DNAemia [62, 63]. Together, these clinical data suggest that MPA use, *per se*, does not increase CMV replication risk.

#### Impact on CMV-Specific Immunity

Among immunosuppressive agents, MPA displays the most selective antiviral actions against CMV *in vitro*. In combination with tacrolimus, MPA demonstrated synergistic lethality against CMV in cell culture models, although these effects occurred at concentrations exceeding those achieved clinically [50].

MPA induces broad suppression of adaptive immunity by depleting CD4^+^, CD8^+^, and CD19^+^ lymphocytes, in part through apoptosis [54]. It downregulates CD25 expression, thereby impairing IL-2–driven T-cell activation and proliferation. *In vitro*, MPA decreases IFN-γ production, not by impairing cytokine synthesis per cell, but by reducing the total lymphocyte pool capable of activation. Consequently, the antiviral arm of the immune response may be dampened in proportion to overall lymphocyte depletion. Recent studies have examined the effects of MPA on CMV-specific T-cell responses. Krueger et al. reported that activation of CD4^+^ and CD8^+^ T cells by CMV pp65 antigen is not significantly affected by MPA alone [51]. MPA had high IC50 values at 3 ng/mL for IL-2. However, MPA strongly reduces the proliferation of purified CMV-specific T cells and γδ T cells *in vitro* [52], indicating a selective antiproliferative effect without full functional suppression. Indeed, Egli et al. showed that MPA induced a moderate reduction in IFN-γ production by CMV peptide–stimulated T cells [33], and Krueger et al. found no significant reduction in IFN-γ–producing CMV-specific T cells upon MPA exposure [51]. Moreover, MPA does not impair cytotoxic T-cell function, since Krueger et al. observed preserved perforin and granulysin production and intact cytolytic activity of CMV-specific T cells exposed to MPA (Figure 2) [51].

In conclusion, MPA has not been shown to independently increase CMV replication or recurrence in SOT recipients. However, it is frequently the first immunosuppressive agent to be reduced or discontinued during CMV infection, mainly because of its myelosuppressive effects, its strong inhibitory impact on T-cell proliferation critical for CMV control, and its contribution to the overall burden of immunosuppression. Reducing or withdrawing MPA may therefore help limit cytopenias and promote immune recovery during CMV infection.

### Corticosteroids

Maintenance steroid dose has been shown to significantly associate with death with a functioning graft due to cardiovascular disease or infection during years 2–5 after kidney transplantation [64]. Glucocorticoids inhibit NK cell cytotoxicity and may impair antigen presentation by reducing the expression of major histocompatibility complex (MHC) class II molecules on circulating monocytes, thereby diminishing T-helper cell activation [65]. In addition, prednisolone (at a high dose of 0.5 μg/mL) exerts broad inhibitory effects on CMV-specific T cells, including decreased activation, reduced IL-2 production, lower frequencies of CMV-specific IFN-γ–producing T cells, decreased production of perforin and granulysin, and inhibition of cytotoxic activity [51] (Figure 2). Consistent with these findings, the 2-year cumulative incidence of CMV infection in simultaneous pancreas–kidney transplant recipients was significantly lower in those managed with a prednisone-free protocol compared to standard steroid-based regimens, particularly among high-risk recipients (D^+^/R^−^ or D^+^R^+^) (18% vs. 36%, p < 0.05) [66]. However, this clinical observation remains isolated in the literature, as subsequent randomized trials did not demonstrate a reduced incidence of CMV infection with rapid steroid withdrawal [67, 68], nor did the 2016 Cochrane meta-analysis confirm such an effect [69].

Although corticosteroids impair immune function, evidence that steroid withdrawal reduces CMV risk remains inconsistent. This discrepancy likely reflects the higher doses used *in vitro* compared with the much lower maintenance doses used clinically.

## The Protective Exception: mTOR Inhibitors

Multiple randomized trials and meta-analyses demonstrated a lower incidence of CMV infection and disease in patients receiving *de novo* mTOR inhibitors compared to CNI-based or MPA-containing regimens [70]. Trials comparing CNI + EVR to CNI + MPA consistently report fewer CMV events in the mTORi groups [71–79]. Prospective studies designed with CMV infection as a primary endpoint confirmed that CMV DNAemia and clinically significant infections were substantially reduced in EVR + low-dose CNI regimens, particularly among CMV-seropositive recipients [47, 77]. Head-to-head comparisons between EVR and sirolimus demonstrated comparable protection against CMV [78]. Even in high-risk patients receiving ATG, EVR was associated with fewer CMV events compared to MPA [71, 79]. Accordingly, the Fourth International Consensus Guidelines for CMV in SOT recipients recommend preemptive or close clinical monitoring in CMV-seropositive kidney transplant patients receiving *de novo* mTOR-based immunosuppression [80].

Data in D^+^/R^−^ kidney transplant recipients remain limited but suggest reduced CMV disease with CNI-EVR compared with CNI-MPA regimens. In a monocentric study, basal and peak viral loads were significantly lower under CNI-EVR [81], consistent with the TRANSFORM study findings [82]. However, other studies found no significant difference in CMV DNAemia [83], warranting further investigation in this subgroup.

Similar reductions in CMV incidence under mTORi therapy have been observed in heart transplantation [84–88], pediatric renal transplantation [87], lung transplantation [88–90], and liver transplantation [91]. Meta-analyses and systematic reviews further confirm that mTOR inhibitors reduce CMV infection rates across various organ types [92, 93].

Conversion to mTOR inhibitors immediately after treatment of a first CMV episode significantly reduces the risk of recurrence in R^+^ kidney transplant recipients [94]. However, a retrospective analysis including heterogeneous cohorts did not show a significant difference in recurrence [95], highlighting the need for prospective data in D^+^/R^−^ high-risk population. Interestingly, in belatacept-treated patients, conversion from MPA to mTORi reduced CMV replication and recurrence [41], supporting the protective effect of mTOR inhibition even in settings of costimulation blockade.

CMV replication depends on both rapamycin-sensitive and rapamycin-resistant mTORC1 activity [96]. mTOR activation supports the translation of viral proteins and the maintenance of protein synthesis during infection [96, 97]. *In vitro*, inhibition of mTORC1 prevents the accumulation of immediate early, early, and late CMV proteins [98]. During late infection phases, sustained mTOR activation is required for synthesis of late viral proteins such as pUL44 and pp65. Rapamycin strongly suppressed CMV replication three to five days post-infection in macrophages, confirming that mTOR is essential for viral replication in myeloid cells [99]. Pre-infection treatment with everolimus suppresses viral spread and reduces the number of infected cells *in vitro* [100]. These findings collectively indicate that mTOR inhibition can directly impair CMV replication, particularly in the late stages of the viral cycle, by interfering with viral protein translation and host cell metabolism.

mTOR plays also a central role in T-cell differentiation and memory formation. mTOR inhibition has been shown to enhance both the quantity and functional quality of virus-specific CD8^+^ T cells, promoting the generation of long-lived memory populations [101, 102]. Under sirolimus monotherapy, antigen-specific CD8^+^ responses are suppressed toward allografts but augmented against viral or bacterial pathogens. *In vitro*, activation of CD4 and CD8^+^ T cells by CMV pp65 antigen is not significantly inhibited by EVR alone [51]. Sirolimus inhibits IL-2 production at low IC_50_ concentrations (∼2 ng/mL) [33], and EVR reduces CMV-specific T-cell proliferation [51]. Nevertheless, *in vivo*, switching from cyclosporine-MPA to EVR-based therapy increases circulating CMV-specific CD8^+^ T-cell counts [103]. These data indicate that mTOR inhibition transiently dampens proliferation *in vitro* but enhances functional antiviral T-cell responses *in vivo* (Figure 2).

**FIGURE 4:**
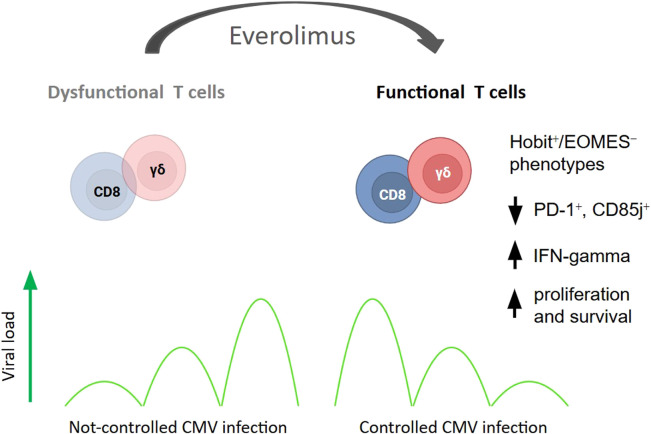
Everolimus restores CMV-specific T-cell functionality and enhances antiviral immunity. mTOR inhibition promotes the proliferation and survival of CMV-specific terminally differentiated effector memory T cells (TEMRA), reduces exhaustion markers (PD-1^+^, CD85j^+^), and shifts transcriptional programs toward a Hobit^+^/EOMES^−^ effector phenotype. Low-dose mTOR inhibitor therapy also enhances IFN-γ production in CMV-specific γδ T cells, thereby supporting effective CMV control.

Sirolimus markedly reduces IFN-γ production *in vitro* [33]. However, EVR does not significantly impair IFN-γ production by CMV-specific T cells stimulated with pp65 antigen [51]. Such discrepancies may reflect differences in experimental conditions or drug concentrations. Indeed, in renal transplant recipients, mTORi therapy has been associated with higher frequencies of IFN-γ–producing CMV-specific cells compared with MPA [104]. Mechanistically, mTOR inhibition converts dysfunctional CMV-specific T cells into functional effector cells, enhancing γδ and αβ T-cell responses and correlating with lower CMV incidence [52]. mTORi therapy improves the proliferation and survival of terminally differentiated effector memory cells (TEMRA), reduces exhaustion markers (PD-1^+^, CD85j^+^), and shifts transcriptional profiles toward Hobit^+^/EOMES^−^ phenotypes. Low-dose mTOR inhibition enhances T-cell receptor signaling and IFN-γ production in γδ T cells when compared to MPA [52]. In the EVER-CMV trial, continuous EVR therapy maintained improved CMV-specific T-cell functionality, while discontinuation led to loss of this benefit [47] (Figure 4).

Furthermore, EVR does not inhibit cytotoxic function: CMV pp65-stimulated PBMCs maintain perforin and granulysin production, and EVR-treated CMV-specific T cells preserve cytotoxicity against infected fibroblasts [51].

Overall, mTOR inhibition represents a dual-action strategy—providing effective immunosuppression while conferring viro-immunologic protection against CMV in R^+^ patients. Nevertheless, mTOR inhibitors are discontinued in approximately one third of patients because of intolerance, which may subsequently re-expose these patients to an increased risk of CMV infection.

## The Cumulative Risk Paradigm

CNI alone or MPA alone have not been shown to independently increase the risk of CMV infection in SOT recipients. Therefore, the increased risk observed is likely attributable to transplant-related factors such as the risk of primary infection in D^+^R^−^ patients and the overall degree of immunosuppression. A prospective study by De Weerd et al. found no statistically significant differences in CMV replication between patients receiving tacrolimus monotherapy and those on a tacrolimus–MPA regimen in low-risk kidney transplant recipients without corticosteroids [105]. However, an earlier study by Shapiro et al. —one of the first to introduce the concept of triple combination regimens to prevent allograft rejection—reported a twofold higher incidence of CMV infection when MPA was added to a tacrolimus/prednisone regimen (8.5% vs. 16.7% at 15 months) [106]. In line with this finding, CNIs appear to affect viral eradication during antiviral therapy. In the VICTOR trial, patients on dual immunosuppressive therapy (versus triple) and those with lower CNI concentrations (≤150 ng/mL for cyclosporine and ≤5 ng/mL for tacrolimus) demonstrated significantly higher rates of viral clearance, with odds ratios of 2.55 and 5.52, respectively [49]. These clinical observations are further supported by recent experimental data from Krueger et al., who showed that activation, IL-2 production, proliferation, IFN-γ secretion, and cytotoxicity of CMV pp65-specific CD4^+^ and CD8^+^ T cells were significantly reduced in the presence of Tacrolimus + EVR + Prednisolone or Tacrolimus + MPA + Prednisolone compared with Tacrolimus or MPA alone [51]. Taken together, the cumulative burden of immunosuppressive therapy, rather than any single agent, appears to drive CMV susceptibility and delayed viral clearance.

## Conclusion

In summary, CMV infection results from the combined impact of immunosuppressive agents on both innate and adaptive immunity. While ATG exert the strongest inhibitory effects on CMV-specific T-cell function, CNIs and MPA contribute mainly through cumulative immunosuppression rather than direct viral promotion. In contrast, mTOR inhibitors show protective antiviral and immunomodulatory properties. Belatacept carries a specific risk of late and atypical CMV disease. Overall, optimizing immunosuppressive balance and incorporating immune monitoring remain essential to prevent CMV complications.
